# Adapting the Crohn’s disease exclusion diet to a Nordic framework: a theoretical approach to cultural and nutritional customization

**DOI:** 10.3389/fnut.2025.1590847

**Published:** 2025-06-19

**Authors:** N. Vivanco Karlsson, R. Sigall-Boneh, K. Mårild, E. M. Hård Af Segerstad

**Affiliations:** ^1^Department of Pediatrics, Skaraborg Hospital, Skövde, Sweden; ^2^Department of Pediatrics, Institute of Clinical Sciences, Sahlgrenska Academy, Gothenburg, Sweden; ^3^Pediatric Gastroenterology and Nutrition Unit, The E. Wolfson Medical Center, Holon, Israel; ^4^Tytgat Institute for Liver and Intestinal Research, Amsterdam Gastroenterology Endocrinology and Metabolism, University of Amsterdam, Amsterdam, Netherlands; ^5^Department of Pediatrics, Institute of Clinical Sciences, Sahlgrenska Academy, Gothenburg, Sweden; ^6^Pediatric Research Institute, Oslo University Hospital, Oslo, Norway; ^7^Clinical Sciences, Lund University, Malmoe, Sweden

**Keywords:** pediatric Crohn’s disease, nutritional treatment, Crohn’s disease exclusion diet, enteral nutrition, Nordic diet

## Abstract

Emerging evidence from diverse populations highlights the therapeutic benefits of the Crohn’s Disease Exclusion Diet (CDED) in managing Crohn’s Disease. To improve the efficacy of the CDED, there is a need to focus on cultural adaptation and individualization to support dietary adherence to this advanced nutritional therapy. This paper provides a theoretical framework for modifying the CDED to align with a Nordic diet (Nordic-CDED), while retaining the core principles and nutritional characteristics of the original protocol. Through a comprehensive literature review and expert consensus, we propose a Nordic-CDED that incorporates European regulations on food additives, the 2023 Nordic Nutritional Recommendations, and Nordic food culture. We present a theoretical foundation for an inclusion of whole foods and discuss the selection of enteral formulas. The adaptation also emphasizes the importance of dietitian-led guidance to tailor the Nordic-CDED to the patient’s age, lifestyle, and personal preferences. Further research is required to assess the clinical effectiveness and adherence to the Nordic-CDED.

## Introduction

### The role of diet in the onset and progression of Crohn’s disease

The global incidence of Crohn’s disease (CD) has risen significantly in recent decades, with the most marked increase in the pediatric population from 20,897.42 cases per 100,000 persons/year reported in year 1990 to 25,658.55 cases per 100,000 persons/year reported in year 2019 (1–3). Northern Europe has one of the highest prevalence rates of inflammatory bowel diseases (IBD) in the world, with CD affecting 0.13% and ulcerative colitis affecting 0.19% of the population (3, 4). IBD is associated with high morbidity, reduced quality of life, and increased healthcare costs, which can also have a significant impact on a patient’s diet and sense of identity (5–8). While the exact mechanisms underlying CD pathogenesis remain unclear, it is believed to involve complex interactions between genetic, immune, microbial, and environmental factors (9, 10).

Recent advancements in both basic and clinical science have significantly broadened our understanding of the role dietary factors play in the development and progression of CD. Emerging evidence suggests that certain dietary components, particularly ultra-processed foods, may trigger and sustain inflammation in CD (11–14). In the 1990s, exclusive enteral nutrition (EEN) was shown to induce remission in mild to moderate active CD. In the Nordic countries, the induction therapy for pediatric patients with active luminal CD, is EEN because of its proven efficacy and feasibility (15). While the underlying mechanisms of EEN remains unknown (15–17), it has been proposed to favorably modulate the gut microbiome (18), the intestinal barrier function and immunity (19). It has also been hypothesized that part of the mechanism by which EEN works may be related to the exclusion of dietary components that are potentially pro-inflammatory (19). However, EEN is challenging to complete due to taste fatigue, poor palatability, nausea and bloating, and the social and psychological impact on the patient (20, 21). Given the onerous nature of EEN, application beyond remission is unfeasible for long-term or maintenance therapy (22). Parents of children with CD previously treated with EEN have expressed a need for a solid food-based alternative (5, 18). However, several studies have shown that partial enteral nutrition (PEN) paired with an unrestricted diet does not yield similar clinical remission rates as EEN (19, 23, 24).

In line with the emerging evidence of CD pathogenesis, a range of effective nutritional therapies for CD has evolved to include whole foods, with the goal of improving dietary adherence and quality of life (11, 16, 18, 25, 26). While most of these dietary therapies have focused on reducing inflammation during active disease (18, 22, 27), they are used for a limited period because of the extensive food restrictions that increases the risk of nutritional deficiency and reduction in quality of life. Unfortunately, within a few weeks of completing these diet therapies, there is a fast regression to the original inflammation status in the bowel (11, 16, 18, 28–30). The Crohn’s Disease Exclusion Diet (CDED), a nutritionally complete diet that combines PEN with whole foods, has emerged as an alternative therapy to EEN with a potential for long-term use. Since 2014 a growing body of evidence has continued to support its use in inducing and potentially maintaining remission in both children and adults with CD (31–38).

This paper aims to develop a theoretical framework for adapting the CDED to a Nordic context. The framework was designed with consideration of European food additives regulations, local food production, and nutritional adequacy, all in an effort to support individualization and promote long-term adherence to the dietary therapy.

## Methods

### Literature review on the core principles of the diet CDED

We conducted a literature review where source selection criteria were applied: studies related to the use of EEN or CDED in patients with CD, children, and adults, encompassing randomized clinical trials, observational studies, meta-analyses, systematic reviews, as well as documents containing recommendations and guidelines. Case studies, articles not published in the English language, and studies involving pregnant and breastfeeding women and athletes were excluded from the review.

The following databases were utilized: PubMed and Google Scholar. The search process employed the following search strings: “IBD and diet therapy,” “Crohn’s Disease Exclusion Diet,” “Food additives and IBD,” “Total Enteral Nutrition,” “Diet adaptation,” “Nordic Diet.” In total, 91 literature papers were collected.

### Expert consensus to develop a theoretical model for adaptation of the CDED to the Nordic setting

We developed this adaptation through a group consensus in collaboration with one of the developers of the original CDED. The fundamental principles of CDED were carefully considered, leading to the development of a Nordic modification. This modified version considered regulations on food additive use in the Nordic food industry, the Nordic Dietary Guidelines, as well as Nordic dietary habits and local foods.

The design of the Nordic-CDED underwent a comprehensive review by the group of authors and was systematically compared with the original CDED. Additionally, a thorough evaluation of other published modifications was conducted, identifying elements that deviated from the original CDED but still resulted in similar clinical effects. These elements were carefully assessed and integrated into the Nordic-CDED where appropriate.

### Core principles of the Crohn’s Disease Exclusion Diet

The CDED aims to exclude dietary components hypothesized to be detrimental to the gut microbiome and intestinal barrier function (13, 14, 19, 36, 39–43). The diet restricts the intake of foods rich in animal fat, red and processed meat, protein sources rich in taurine, gluten and wheat protein, as well as food additives including maltodextrin, emulsifiers, artificial sweeteners, carrageenan, and sulfites (44). The exclusion of ultra processed food is one of the most important elements of CDED and exclusion of additives based on the Israeli Food Control Services regulations (44). Moreover, the CDED includes specific fruits and vegetables that provide various dietary fibers and resistant starch, which serves as substrates for short-chain fatty acid (SCFA) producing bacteria (45, 46).

The CDED is a standardized diet consisting of 3 phases (Table 1) (36). The first phase (weeks 0–6) is highly restrictive, excluding all potential triggering ingredients, while emphasizing consumption of high-quality protein sources and microbiome-enhancing ingredients. In this phase, 50% of the energy requirements is provided by PEN, and the remaining 50% from whole foods. The diet is liberalized for weeks 6–12 (phase 2), enabling a gradual introduction of previously restricted foods and a reduction of PEN (25%). Phase 3 (weeks 13–18) permits up to twice a week, the inclusion of most unprocessed foods, along with the consumption of products containing food additives (38). Since there is limited evidence of excluding foods or additives beyond phase 3, it is advised that the patient adopts an overall healthy diet in line with general dietary guidelines after completion of Phase 3 (38).

**Table 1:** Description of the three phases of Crohn’s disease exclusion diet*.

Phase	Duration	Food Intake	Enteral nutrition
		% of daily energy requirements	
1	6 weeks	50%	50%
2	6 weeks	75%	25%
3	6 weeks	75%	25%

*Levin et.al (36).

The CDED was designed to be adaptable across various countries and cultures, incorporating simple and internationally accessible foods, but was mostly focused on “Western” populations (26, 47, 48). It recommends chicken and egg as the main sources of protein, potatoes as the main starch source, and apples and bananas to provide soluble fiber and pectin. Additionally, it includes a list of allowed whole foods to ensure nutritional adequacy. Table 2 outlines the recommended and excluded foods, along with the rationale for their inclusion or exclusion in the various phases of the CDED.

**Table 2:** Recommended and excluded foods in Crohn’s disease exclusion diet (CDED): rationale for selection*.

Suggested function	Phase 1		Phase 2	Phase 3
	Food	Rational of inclusion	Added Food	
Protein source	Fresh chicken breast	Low fat	Canned tuna	Seafood
		Low taurine	Low-fat fresh fish	Salmon
	Egg	Low fat		Yoghurt
Gut microbiota	White rice and rice-based foods	Resistant starch	Bread (with gluten)	Pasta
	Green banana	“	Sweet potato	
	Potato	“	Lentils, chickpeas, beans	
	Apple (peeled)	Pectin	Quinoa	
Intestinal integrity	Avocado	Unsaturated fat	Oatmeal	
	Vegetable oil (olive/rapeseed)	“	Peach, pear, kiwi	All fruits
	Cantaloupes	Low in insoluble fiber	Blueberry	
	Strawberries	“	Zucchini	All vegetables
	Salad	“	Champignon	
	Spinach	“	Broccoli	
	Tomato	“	Cauliflower	
	Carrot	“		
	Cucumber	“		

*Levin et.al (36).

The enteral formula used in CDED’s first trials was Modulen IBD, specifically developed for patients with IBD (31, 36). This polymeric formula is rich in Transforming Growth Factor Beta (TGFβ), a potent anti-inflammatory cytokine. In murine models, TGFβ has demonstrated a positive effect on lesions associated with chronic inflammation (49, 50).

### Rationale for a modified CDED: enhancing adherence and individualization

Restricted eating because of gastrointestinal symptoms is common in IBD patients and increases the risk of nutritional deficiencies and malnutrition (6, 7, 51). In various reports, nearly half of pediatric and adult IBD patients indicate that they avoid or limit their intake of fruits and vegetables because of symptom flare-ups (7). Patients with IBD use different food-related strategies to control symptoms, such as identifying and avoiding triggering foods, following restrictive diets, controlling portion size, and eating more or less frequently, which may potentially have consequences on their nutrient intake and status. Such maladaptive behaviors, combined with limited knowledge about diet in IBD, may adversely affect patients food-related quality of life and result in social isolation (52). In addition, suboptimal nutrient intake can in turn negatively impact their clinical prognosis (7, 53, 54). Furthermore, as seen in other chronic conditions such as celiac disease and food allergies, extensive exclusion diets are associated with an increased risk of maladaptive eating behaviors (55). One such condition is Avoidant/Restrictive Food Intake Disorder (ARFID), where dietary intake is significantly limited for reasons unrelated to body image, including fear of adverse physical reactions, perception of pain, or a general lack of interest in eating (38). The development of ARFID can also contribute to social anxiety and withdrawal, further impacting quality of life (56, 57).

The primary goal of any nutritional therapy is to optimize the patient’s nutritional and clinical status while supporting growth and development (26). This can be achieved by tailoring the therapy to the individual patient’s needs, while simultaneously improving dietary adherence. Since diet is a central aspect of daily life and modifying eating habits can be challenging (58–60), it is crucial to guide patients in adopting not only a clinically effective diet, but it should also be manageable, socially acceptable, and sustainable (6, 56, 61). It is essential to develop evidence-based nutritional therapies that consider adaptation and individualization, with the goal of supporting long-term healthy dietary habits (58).

Recent clinical studies on the implementation of a modified CDED in various populations exemplifies how this nutritional therapy has been adapted to diverse food cultures, religious practices, and individual nutritional needs, while maintaining alignment with national dietary guidelines and achieving improved adherence (33–35, 37, 61, 62). These adaptations have showed improved individualization, acceptability, and adherence to the diet (33, 34, 63). To date, data on adaptations of the CDED within the Scandinavian settings are limited, and its clinical application remains scarce. In our clinical practice, 10 pediatric patients have undergone CDED, with generally good adherence. However, in most cases, modifications were necessary, including an expanded list of permitted foods and enteral products to improve feasibility and patient acceptance.

### Adapting the CDED to a Nordic context

The Nordic diet is characterized by native berries, legumes, apples, pears, root vegetables, cabbage, cauliflower, curly kale, and mushrooms, as well as whole grains such as barley, wheat, oats, buckwheat, and rye. It also includes regular fish consumption, seaweed, and free-range animal products (64, 65). Given these core foods in the Nordic diet, it is theoretically possible to incorporate a wider variety of foods across the different phases of the CDED, while still maintaining its presumed anti-inflammatory benefits and ensuring adequate nutritional intake. Table 3 outlines core foods in the Nordic diet. In the Nordic countries, aligned with the fundamental principles of the CDED, additional gluten-free carbohydrate sources, such as buckwheat and arrowroot, are included.

**Table 3:** Key foods in the Nordic diet.

Nordic foods	Examples of foods*
Fruits & berries	Rose hip, blueberry, lingonberry, apple, pear, prune
Vegetables	Cabbage, cauliflower, brussels sprouts, broccoli, fennel, spinach, sugar peas, kale
Root vegetables	Onion, kohlrabi, turnip, carrot, parsnip, beetroot, viper’s grass
Nuts	Almonds
Legumes	Brown beans, yellow peas, green peas
Meat	Beef, pork, lamb, reindeer, sausage
Poultry	Chicken, turkey
Dairy products	Low-fat or fermented milk, cheese
Fish	Herring, Baltic herring, mackerel, salmon
Eggs	Hen
Cereals	Whole grain rye, whole grain wheat, oat bran, barley flakes, muesli, pearled barley
Seeds	Linseed, psyllium, sunflower seeds
Fats & Oils	Vegetable fat spread, vegetable liquid margarine, sunflower, linseed, and rapeseed oil
Sweets	Baked goods, jam based on Nordic fruits and berries

*Bere and Brug (65), Krznari´ et al. (80).

The Nordic diet further includes a variety of fruits and vegetables rich in fiber and starch, such as plums and carrots, as well as fresh fish, seafood, and sources of unsaturated oils, such as almonds. Table 4 presents the whole foods recommended in the original CDED, along with suggested additions for a Nordic adaptation.

**Table 4:** Crohn’s disease exclusion diet (CDED) recommended whole food and suggested additions in a Nordic adaptation.

Suggested function	CDED	Characteristics	Additions in the Nordic-CDED
Protein source	Fresh chicken breast	Low fat/ Taurine	Fish and fresh sea foods
	Egg		Natural yoghurt (without food additives)
			Tofu (locally produced)
			Texturized soy protein
Gut microbiota	White rice and rice products	High-resistant starch content	Gluten-free oats (free from wheat starch if of labeled)
	Green Banana		
	Potatoes		
	Apple	High pectin content	Gooseberries
			Plums
			Parsnip
			Cherries
		Gluten-and wheat free	Naturally gluten-free grains and flour (buckwheat, arrowroot, sorghum, quinoa, rice, gluten-free oats and potatoes).
Intestinal membrane	Avocado	Vegetable fat	Almonds butter
	Vegetable oil		Vegetable oil (olive, avocado, rapeseed-canola)
	Fruits and berries	Low fiber content	Fruits and berries
	Cantaloupe		Plums
	Honeydew		
	Strawberries		
	Vegetables		Vegetables
	Carrot		Zucchini
	Salad		Rucola
	Spinach		Sugar peas
	Tomato		
	Cucumber		
	Maple syrup	Simple Carbohydrates	Sweets without food additives
	Honey		

### Food additives in the Nordic CDED

The European Food Safety Authority (EFSA) sets regulations for food additives permitted in the food industry in the European Union (66). These regulations are enforced across all Nordic countries and are stricter compared to those in Israel (44). For example, additives such as sulfites, carrageenan, and carboxymethylcellulose are allowed in fewer products under EFSA regulations compared to in Israel. Furthermore, while Israeli regulations permit additives like titanium dioxide, EFSA banned its use in 2022 (44). These regulatory differences present an opportunity to potentially expand the range of whole foods allowed the Nordic CDED. Table 5 lists the excluded elements of the CDED including several food additives, along with examples of foods that may contain them according to EFSA regulations (44, 66).

**Table 5:** Excluded elements of the Crohn’s disease exclusion diet (CDED) and examples of containing foods according to the European food safety authority (EFSA).

Excluded element	E-number	Allowed for inclusion in foods
High animal fat, taurine		Red meat/turkey
Gluten/wheat protein		Wheat, rye, and barley
Saturated fat		Dairy
Artificial sweeteners	E 950, 951	Juices and soda
Sulphites	E221-228	Dried fruit
Titanium dioxide	E 171	Powdered food
Emulsifiers		Processed food
Polysorbate 80*	E433	Ice cream, confectionery, chewing gum, soups, sauces, slimming products, desserts, milk and cream-like products and dietary supplements.
Carboxymethylcellulose*	E466	May be used without quantity limitation in most foods.
Maltodextrin***		Pasta, cooked cereals and rice, meat substitutes, baked goods, salad dressing, frozen ready meals, soups, sugar, confectionery, energy and sports drinks. Thickener and preservative, including
Carrageenan**	E407	Desserts, ice cream, milkshakes, yoghurt, condensed milk, sauces, pâtés, plant-based drinks

*Miyazato et al. (39), Borsani et al. (41), Chassaing et al. (68).

A key limitation in current research on food additives is that much of the supporting evidence comes from animal models and human cell lines (19). It is still unclear if studies translate to human physiology and the specific quantities that may induce harm (33). Additionally, as there is yet no conclusive evidence regarding the mechanisms, amounts, and types of certain food additives, much remains to be defined in the CDED (19, 32, 33, 67, 68).

### Nutritional recommendations

The original CDED (36) was initially developed with mandatory whole food in suggested quantities, alongside PEN. To be nutritionally balanced, specifically for young children, the amounts of included whole foods should be adjusted, as pre-determined quantities may exceed recommended daily intakes of protein, vitamin A, and Iron (69). In further developments and modifications to the CDED, the individualization of specific amounts of whole foods has been emphasized in this regard (38, 61). According to the European Society of Parenteral and Enteral Nutrition (ESPEN), the energy and nutrient requirements for children with IBD are generally consistent with those of the general population, although specific complications may necessitate adjustments to certain nutrients (70). In the Nordic-CDED, we suggest to use age-and sex-specific recommendations when determining the appropriate amounts of the recommended whole foods, particularly for protein intake (69). Table 6 outlines the phases of the Nordic-CDED and suggested recommended daily intake of energy and macronutrients.

**Table 6:** Energy and nutrient recommendations for the three phases of the Nordic Crohn’s disease exclusion diet.

Phase	1	2	3
Energy	In line with recommended*, adjusted to age and sex		
Enteral Nutrition (oral or tube feeding)	50% of total energy requirement	25% of total energy requirement	25% of total energy requirement
Protein	In line with recommended*, adjusted to age and sex 10–20% of energy intake		
Carbohydrates	45–60% of total energy intake*		
Fiber	Low intake of fiber adjusted to age* Adults: < 3 g/MJ of energy intake/day Children: < 2–3 g/MJ energy intake/day		In line with recommended for age* Adults: 3 g/MJ of energy intake/day Children: 2-3 g/MJ energy intake/day
Fat	25–40% of total energy intake*		
Saturated	Low intake		< 10% of energy intake

*Blomhoff et al. (69).

### Choice of enteral nutrition or oral nutritional supplementation

A deteriorated nutritional status is common in Crohn’s disease. Children are a specifically vulnerable group as poor nutritional status affects growth and delays overall development (71). While EEN is often used as induction therapy in CD, a key secondary clinical goal is to support nutritional rehabilitation, and growth in children. This aligns with the rationale for using PEN as a maintenance therapy to ensure long-term nutritional sufficiency (35, 70). In clinical practice, there is considerable variation in the types of formulas used for EEN. Several publications, including one that examined 61 commercial enteral formulas and oral nutritional supplements used in EEN for CD (72), have demonstrated similar clinical efficacy across different formulas, regardless of the level of protein hydrolysis (polymeric, semi-elemental, or elemental), the range of food additives included, or nutrient concentration (15, 62, 70, 71).

Modulen IBD, a polymeric formula based on milk (protein and fat), that contains added soy lecithin, has been the formula of choice in most CDED trials (31, 36, 73–75). However, several studies have reported similar clinical outcomes of the CDED using other enteral formulas (31, 34, 35, 62). Most of the commonly used nutritional supplements contain food additives such as maltodextrin and emulsifiers. When adapting the Nordic-CDED, we suggest selecting an enteral formula or oral nutritional supplement that has shown a clinical effect in previous trials. Furthermore, factors such as availability, cost, and palatability should be carefully considered, as they play a crucial role in individualizing the diet and ensuring long-term adherence (5, 71, 76).

## Discussion

This article presents the theoretical rational for an adaptation of the CDED to a Nordic modification. A key aspect of this adaptation involves a comprehensive review of the core principles and rationale of the original CDED. The Nordic-CDED presented is based on these core principles and proposes additional allowed whole foods, typical for the region. Furthermore, the modified Nordic-CDED incorporates current EFSA regulations on food additives and aligns with the Nordic Nutrition Recommendations to tailor nutritional requirements to individual needs. The primary goal of this adaptation is to support the individualization of the CDED. However, this theoretical framework must be validated in clinical trials to assess its clinical efficacy and impact on patient experience compared to previous findings (74). Such trials should evaluate remission rates, using objective measurements of inflammation and endoscopic outcomes, nutritional adequacy, dietary adherence, and patient-reported outcomes on quality of life and diet satisfaction.

For several decades, EEN was the sole nutritional treatment option for CD. Its efficacy has been well-documented across numerous populations and clinical settings (21, 28, 77). However, significant barriers to its use remain, primarily related to the monotonous and unpalatable taste of enteral formulas, the strict dietary restrictions required, and associated adverse effects such as nausea, vomiting, constipation, and a typically short-lived therapeutic effect (34). To address these limitations, the CDED was developed as an alternative nutritional strategy. By incorporating selected whole foods, many of the challenges associated with EEN can be considerably reduced. CDED has since been implemented in a variety of countries and cultural contexts, highlighting the importance of adapting the diet to local eating habits and food availability (59). Such adaptations aim to mitigate risks commonly associated with restrictive dietary therapies, including disordered eating behaviors, incomplete or unbalanced diets, social isolation, diminished quality of life, and reduced enjoyment of food. Furthermore, overly restrictive diets may heighten anxiety related to eating, fear of adverse events, or concerns about disease relapse factors that CDED seeks to address through a more flexible and sustainable approach (59).

Previous publications have reported on regional modifications to the CDED (33, 34). The Dietitians Crohn’s and Colitis Australian Network (DECCAN) modified the CDED for adults with CD, aligning it with Australian food groups and micronutrient recommendations. They also developed an optimal care pathway for clinical use of the CDED, which included consensus statements, a clinician toolkit, and a patient education material (33). Additionally, in Slovenia researchers compared the effects of a modified CDED versus EEN. In this modification, 75% of the energy requirement was provided by PEN using an alternative enteral formula, with the remaining 25% sourced from food (one meal per day). The Slovenian CDED included regional and locally produced fruits and vegetables, white meat, fish, and added buckwheat and millet as carbohydrate sources. After 6 weeks on the diet, both groups demonstrated similar clinical remission rates, but the CDED group showed a higher endoscopic response compared to the EEN group (34). These studies propose that the original CDED’s clinical efficacy can be maintained with modifications, such as an expanded selection of foods and the use of other enteral products beside Modulen.

As the underlying mechanisms of CDED are not yet fully understood, it is essential that any adaptations remain aligned with its core principles. There is a risk that modifications deviating too far from the original protocol may compromise its clinical efficacy. Therefore, it is crucial to systematically evaluate the impact of each adaptation to ensure that the therapeutic benefits are maintained. Any adaptation of the CDED should offer evidence-based clinical guidance on the exclusion and selection of foods, while considering the various challenges patients may face. It may be beneficial to screen patients starting nutritional therapy to identify those at risk of developing an unhealthy relationship with food. A food-based dietary therapy that provides adequate energy and nutrients could help prevent nutritional deficiencies while promoting growth and development. A clinically effective diet that reduces the risk of inflammatory flare-ups and is also accepted by patients to use long-term would support overall clinical care of patients with CD.

However, every nutritional therapy comes with potential side-effects, including clinical, nutritional, and psychological complexities that must be carefully managed to promote adherence and minimize nutrition-related complications and disordered eating (33). For many patients and their families, diet profoundly affects daily life, including school or work and social interactions (52). Therefore, it is crucial to consider the patient’s psychological state, as well as their religious, cultural, and personal practices, as these factors can influence their relationship with food and, ultimately, their overall quality of life (5, 38, 52). In this, the dietitian plays a crucial role in the success of nutritional therapy (78, 79). In pediatric patients with CD, it is essential to assess the suitability of the specific therapy, tailoring the diet to the patient’s clinical and nutritional status while also considering their preferences and quality of life (61). Effectively communicating dietary advice and adapting the therapy based on current evidence is essential. If successful, the dietitian can help the patient adopt a diet that not only addresses the disease during a flare but also has the potential to become part of a long-term lifestyle that supports gut health and reduces the risk of future flare-ups (52).

Establishing evidence-based long-term dietary therapy for IBD not only offers a potential to reduce the need for immunosuppression and lower the risk of associated side effects, but it may also serve as a bridge between medications and provide a widely accessible and cost-effective treatment option. It is important to continue developing CDED as a nutritional therapy, as a modern CDED diet could serve as a valuable long-term complement to medical treatment for Crohn’s disease.

## Conclusion

We present an adaptation of the original CDED to a Nordic setting, with a theoretical framework and practical guidance aligned with updated nutritional guidelines. The adaptation incorporates a wider variety of recommended foods and guidance on individualizing the diet for CD patients. Moreover, the adaptation includes recommendations to choose enteral formulas to be used. Future research should focus on clinical studies to assess the efficacy of the Nordic-CDED, in comparison with the CDED.
